# Comparison of Myelotoxicity and Nephrotoxicity Between Daily Low-Dose Cisplatin With Concurrent Radiation and Cyclic High-Dose Cisplatin in Non-Small Cell Lung Cancer Patients

**DOI:** 10.3389/fphar.2020.00975

**Published:** 2020-06-26

**Authors:** Zulfan Zazuli, Renate Kos, Joris D. Veltman, Wilma Uyterlinde, Cristina Longo, Paul Baas, Rosalinde Masereeuw, Susanne J. H. Vijverberg, Anke-Hilse Maitland-van der Zee

**Affiliations:** ^1^ Department of Respiratory Medicine, Amsterdam UMC, University of Amsterdam, Amsterdam, Netherlands; ^2^ Department of Pharmacology-Clinical Pharmacy, School of Pharmacy, Bandung Institute of Technology, Bandung, Indonesia; ^3^ Department of Thoracic Oncology, The Netherlands Cancer Institute/Antoni van Leeuwenhoek Hospital, Amsterdam, Netherlands; ^4^ Division of Pharmacology, Utrecht Institute for Pharmaceutical Sciences, Utrecht, Netherlands

**Keywords:** cisplatin, low dose, radiotherapy, myelotoxicity, bone marrow suppression, nephrotoxicity, toxicity

## Abstract

**Aim:**

Antineoplastic effect of cisplatin, the first line treatment in non-small cell lung cancer (NSCLC), is hindered by its nephrotoxicity and myelotoxicity. Both low-dose and high-dose regimens are used in the management of NSCLC. The aim of this study is to assess the risk on myelotoxicity and nephrotoxicity from the daily low-dose cisplatin (DLD) treatment as compared to cyclic high-dose cisplatin (CHD).

**Methods:**

A retrospective cohort study was conducted. NSCLC patients treated with cisplatin between 2011 and 2018 in the Amsterdam UMC or Antoni van Leeuwenhoek cancer hospital were studied. Myelotoxicity and nephrotoxicity were defined based on common terminology criteria (CTCAE v4.03) and categorized as ≥grade 1 and ≥grade 2. Modified Poisson regression and Cox proportional hazards model were used to estimate relative risks and cumulative hazard respectively.

**Results:**

Of the 115 NSCLC patients receiving DLD (N=62) and CHD (N=53), 60% had ≥grade 1 anemia, 33.9% leukopenia, 31.3% neutropenia, 27.8% thrombocytopenia, 32.2% acute nephrotoxicity with combined definition (Cr-electrolyte nephrotoxicity), and 58.3% chronic nephrotoxicity. The DLD group was older, had an earlier cancer stage, had more comorbidities, and had higher baseline albumin levels. In the DLD group less ≥grade 2 toxicities were reported compared to the CHD group except for Cr-electrolyte nephrotoxicity. However, there was a stronger association in the DLD group with ≥grade 1 leukopenia, thrombocytopenia, and Cr-electrolyte nephrotoxicity. The DLD group developed significantly more ≥grade 1 leukopenia [adjusted relative risk (adjRR)=1.83, 95% CI 1.02–3.27], thrombocytopenia (adjRR=3.43, 95% CI 1.64–7.15), and ≥grade 2 Cr-electrolyte nephrotoxicity (adjRR=3.02, 95% CI 1.20–7.56). The DLD group had a lower adjusted cumulative hazard for developing ≥grade 2 myelotoxicity and chronic nephrotoxicity but not for Cr-electrolyte nephrotoxicity [adjusted hazard ratio (adjHR)=3.90, 95% CI 1.35–11.23]. In contrast, DLD showed protective effect to ≥grade 2 nephrotoxicity when definition was restricted to the traditional creatinine-based definition (adjRR=0.07, 95% CI 0.01–0.86; adjHR=0.05, 95% CI 0.01–0.56).

**Conclusions:**

Overall, the DLD regimen was safer than the CHD regimen when assessing the risk of ≥grade 2 myelotoxicity and nephrotoxicity. However, this might not be the case in patients with a higher risk of electrolyte abnormalities.

## Introduction

Cisplatin is the current first line of treatment in locally advanced non-small cell lung cancer (NSCLC); (Vansteenkiste et al., 2013; Besse et al., 2014; Fennell et al., 2016) it acts by creating DNA cross-links that, after failed DNA repair, lead to cell apoptosis (Dilruba and Kalayda, 2016). Cisplatin sensitivity and resistance are patient dependent (Rose et al., 2014), and cisplatin can cause multiple side effects including ototoxicity, nephrotoxicity, and myelotoxicity (Hartmann and Lipp, 2003). A new generation of drugs, including carboplatin and oxaliplatin, is currently available on the market as well. While these drugs reduce the risk of neurotoxicity and ototoxicity, they are known to increase myelotoxicity and are less effective in improving overall survival (Dilruba and Kalayda, 2016). Therefore, despite these new developments, cisplatin is still the standard treatment for lung cancer (Vansteenkiste et al., 2013; Besse et al., 2014). The major dose-limiting toxicities of cisplatin are nephrotoxicity and myelotoxicity. The severity of nephrotoxicity is directly proportional to the amount of cisplatin accumulation taking place in the kidneys (Moon et al., 2011). Nephrotoxicity-indicated by high serum creatinine, low estimated glomerular filtration rate (eGFR), and/or electrolyte abnormalities, appears in about one-third of cisplatin treated patients although percentage may be higher in elderly and children (Pabla and Dong, 2008; Zazuli et al., 2018). Myelotoxicities appear in 16–40% of the patients treated with cisplatin, of which leukocytopenia and neutropenia have the highest incidence (Atmaca et al., 2013; Yu et al., 2015).

Two different forms of cisplatin-based regimens are known to be used in the management of NSCLC: the standard regimen–cyclic high-dose (CHD) cisplatin and the alternative regimen–daily low-dose (DLD) cisplatin regimen with concurrent radiotherapy where cisplatin acts as a radiosensitizer. The current standard, CHD regimen, is chemotherapy, or sequential chemoradio therapy, of two to four cycles with a three to 4 week interval between chemotherapy cycles, each cycle receiving a relatively high cisplatin dose of 75–80 mg/m^2^ (Vansteenkiste et al., 2013; Besse et al., 2014). An alternative regimen, the DLD regimen, is often used in patients with multiple comorbidities. This regimen includes daily low-dose cisplatin (6 mg/m^2^) with concurrent radiotherapy (up to 66 Gy, divided in 24 x 2.75 Gy) (Milano et al., 1990; Schuster-Uitterhoeve et al., 1996). The DLD regimen was shown to have similar efficacy compared to the standard treatment regimen (Takata et al., 2002; Uitterhoeve et al., 2007; Kappers et al., 2011; van Diessen et al., 2016). Furthermore, DLD therapy has a similar, possibly more favorable, radiotoxicity profile, as compared to the highly toxic concurrent high-dose therapy (Biedermann et al., 2000; Belderbos et al., 2007; Uitterhoeve et al., 2007; Kappers et al., 2011).

While other studies have compared the effectiveness and overall safety between both regimens (Belderbos et al., 2007; Uitterhoeve et al., 2007), none have compared DLD and CHD to investigate specific differences in risk for myelotoxicity and nephrotoxicity. Such comparison could provide information needed to support personalized therapy in NSCLC patients.

Thus, we aimed to assess if the occurrence, severity, and time to the development of myelotoxicity and nephrotoxicity differed between patients receiving a DLD regimen and those receiving a CHD regimen. We hypothesized that the DLD regimen is safer than CHD regimen. This study could provide new insights for cisplatin risk management in NSCLC patients, hopefully improving the patients’ quality of life during and after treatment by minimizing the adverse events without compromising efficacy by applying more personalized treatment.

## Methods

### Study Design and Population

We conducted a retrospective cohort study. NSCLC patients were treated with the DLD and CHD regimen at the Amsterdam UMC tertiary care center [location Academic Medical Center (AMC) and Vrije Universiteit medical center (VUMC), between January 2016 and June 2018] or with DLD at the Antoni van Leeuwenhoek cancer hospital (the Netherlands Cancer Institute, between January and 2011 and August 2012) (Uyterlinde et al., 2014). These time windows were selected to ensure uniformity of DLD treatment in terms of prehydration protocol between both centers. To our knowledge, no consistent evidence confirmed the effect of cancer clinical staging to chemotherapy toxicity, especially cisplatin. Thus, we included all patients receiving the two regimens regardless the clinical stage. Patients with an adapted diagnosis of small cell lung cancer (SCLC) or another primary malignancy were excluded.

Cohort entry was defined as the first day of cisplatin treatment. Patients were followed until the date of the adverse event, lost-to-follow-up (treatment discontinuation and/or switching), or the study end date, i.e., 90 days after the last administration of cisplatin to allow for reporting of lagged adverse drug effects, whichever occurred first.

The ethics committee of AMC, has evaluated the protocol (W18_003 # 18.014) and stated that this study was outside the boundaries of the Medical Research Involving Human Subjects Act (WMO).

### Data Collection

The following data was extracted from the electronic patient record: age at start of treatment (years); gender; ethnicity; body mass index (BMI, kg/m^2^); body surface area (BSA, m^2^); cancer stage (I–IV); histology of lung cancer (adeno; squamous; large cell; other); Karnofsky performance status (KPS, score); World Health Organization performance status (WHO PS); date of treatment start; type and dose (mg/m^2^) of chemotherapy received; pretreatment received; amount of hydration received; treatment completion; date of last follow-up; number of chronic comorbidities. Furthermore, we collected lab values at baseline, throughout treatment duration (every week at day 5 for DLD and every cycle 1 day before CHD administration), at the end of treatment, and at 90 days after the last administration of chemotherapy. The following lab values were recorded: creatinine (mg/dl); eGFR by applying Chronic Kidney Disease-Epidemiology Collaboration (CKD-EPI) equation (ml/min/1.73 m^2^); magnesium (mg/dl); potassium (mEq/L); phosphate (mg/dl); albumin (g/dl); bilirubin (mg/dl); hemoglobin (g/dl); leukocytes (cells/mm^3^); neutrophils (cells/mm^3^); and thrombocytes (cells/mm^3^).

### Outcomes

Primary endpoints were cisplatin-related toxicity such as the development of myelotoxicity and nephrotoxicity during the follow-up period. Myelotoxicity consisted of anemia, leukopenia, neutropenia, and thrombocytopenia. Nephrotoxicity consisted of acute nephrotoxicity with combined definition (serum electrolytes and creatinine-based acute kidney injury; Cr-electrolyte nephrotoxicity) and chronic nephrotoxicity (eGFR-based chronic kidney disease). Each toxicity was graded according to the common terminology criteria of adverse events (CTCAE v4.3) (NCI, 2010), for which the criteria of acute nephrotoxicity consisted of the combined criteria of acute kidney injury and electrolyte disorders (hypomagnesemia, hypokalemia, hypophosphatemia) to increase sensitivity of detecting renal tubular injury caused by cisplatin. Beside using modified acute nephrotoxicity definition, we also analyzed the traditional serum creatinine-based acute nephrotoxicity (SCr-AKI) and electrolyte abnormalities separately. We did not collect urine volume data since it was not required by CTCAE criteria. Two cut-offs for adverse events grades were used: ≥grade 1 (overall toxicities) *vs*. no toxicity and ≥grade 2 (moderate-to-severe toxicities) *vs*. no toxicity and grade 1 (mild toxicities). Our secondary endpoint was the kidney function trend marked by change in eGFR over time. Blood tests provided objective measurement and were routinely measured and documented in the clinics. Thus, our investigation focused on myelotoxicity and nephrotoxicity. Although very relevant, other cisplatin-related toxicities e.g., nausea/vomit and neurotoxicity were not regularly measured, making it more susceptible to bias in the setting of such retrospective study.

### Treatment

The DLD group treatment plan was specified as receiving 6 mg/m^2^ of cisplatin for 24 days (24 administrations, 5 times per week), while also receiving intensity modulated radiotherapy up to 66 Gy (24 x 2.75 Gy) 1–2 h after cisplatin administration, the standard low-dose regimen in the hospital. The CHD treatment plan was specified as receiving 30–80 mg/m^2^ cisplatin-based chemotherapy for a three to 4 week interval for 2–3 cycles in combination with pemetrexed (500 mg/m^2^), etoposide (100 mg/m^2^), docetaxel (75 mg/m^2^), or gemcitabine (1.250 mg/m^2^).

### Confounders and Covariates

**Figure 1:**
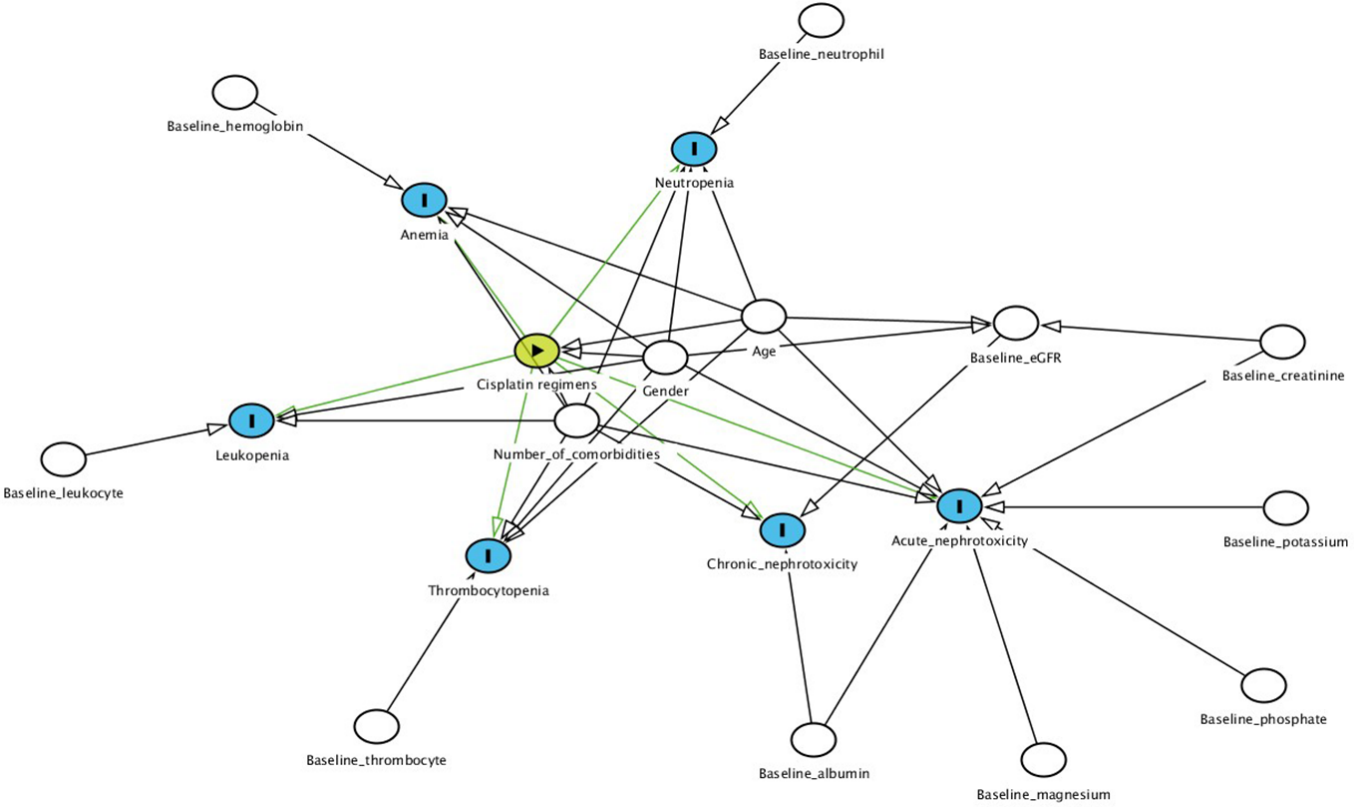
Directed acyclic graphs of covariates included in this study.

The possible confounders that were used as covariates in our models were identified based on directed acyclic graphs (Textor et al., 2016) and were different for each study outcome (**Figure 1**). Age at treatment and gender were covariates for all outcomes (Hardy et al., 2010) except for chronic nephrotoxicity as those variables were already taken into account when calculating individual eGFR. Additional covariates for myelotoxicity were as follows: number of comorbidities, and any of the following baseline disease states as defined by blood cell counts; anemia by hemoglobin, leukopenia by leukocytes, neutropenia by neutrophils, thrombocytopenia by platelets (Moreau et al., 2009; Hardy et al., 2010; Castelan-Martinez et al., 2016; Nishikawa et al., 2017). Number of comorbidities, baseline serum creatinine, albumin, magnesium, phosphate, and potassium were included for Cr-electrolyte nephrotoxicity while baseline albumin and eGFR were covariates for chronic nephrotoxicity (Kidera et al., 2014; Yamamoto et al., 2017). Unlike Cr-electrolyte nephrotoxicity, SCr-AKI was not corrected for baseline electrolyte values while electrolyte abnormalities weren’t corrected for baseline serum creatinine.

### Statistical Analysis

Missing values were analyzed before conducting multiple imputation. Multiple imputations (N=5) were used to address missing data in baseline laboratorium values: albumin (13.04% missing values), serum creatinine (0.87%), magnesium (47.83%), potassium (6.09%), hemoglobin (1.74%), leukocyte (0.87%), neutrophil (16.52%), platelet (1.74%).

Modified Poisson regression models (Zou, 2004) were used to estimate relative risks (RR) comparing the DLD and CHD treatment groups for different outcome cut-offs (≥grade 1 and ≥grade 2 toxicities) adjusted for measured confounders and covariates. We used a generalized estimating equations (GEE) approach to the linear model to compare eGFR from baseline, at the end of treatment and 90 days after the last administration of cisplatin between both treatment regimen, adjusted for number of comorbidity and baseline albumin.

A time-to-event analysis was performed in order to assess whether the time to toxicity development differed between regimens. Only moderate-to-severe adverse events were analyzed to capture clinically relevant adverse events. The Cox proportional hazards model with time frame 120 days after the start of treatment was used to estimate cumulative hazard on occurrence of an event over time (HR) due to differences in treatment duration between groups, adjusting for the same covariates as the modified Poisson regression models. To test the appropriateness of selecting 120 days-cut off, we compared occurrence of first toxicity before and after 120 days since the start of treatment in both treatment arms. The proportional hazards assumption was verified visually by comparing Kaplan-Meier curves of both treatment arm. All statistical analysis was performed with SPSS (version 25; SPSS Inc., Chicago, IL).

## Results

### Subject Characteristics

**Figure 2:**
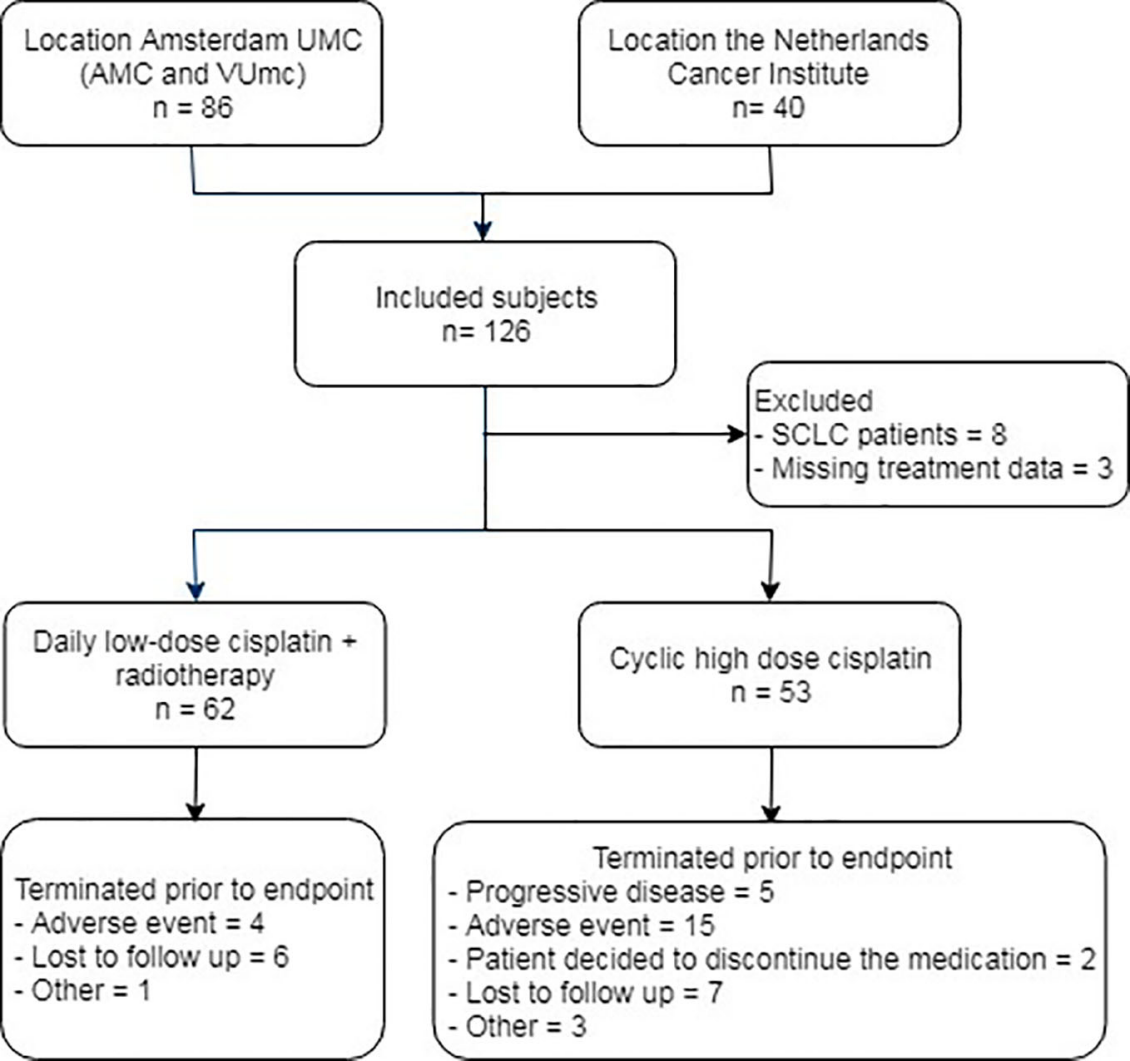
Flow diagram of subject inclusion. AMC, Academic Medical Center; VUmc, Vrije Universiteit medical center; SCLC, small cell lung cancer.

Between January 2016 and June 2018, 86 patients were treated with cisplatin chemotherapy for the indication of non-small cell lung cancer at the Amsterdam UMC: 22 with DLD and 64 with CHD, 49 at location AMC and 37 at location VUmc. In total, 40 DLD cisplatin-treated patients from the Netherlands Cancer Institute between January and 2011 and August 2012 were included in the analysis (**Figure 2**). There were no significant differences in clinical characteristics between DLD-treated patients from Amsterdam UMC and the Netherlands Cancer Institute except in performance status (functional impairment). Overall, patients at the Netherlands Cancer Institute had a better performance status; 67.6% had a WHO performance status of 0, compared to only 36% of the patients treated at Amsterdam UMC (**Table S1**). In total 11 patients were excluded from the study: eight patients had an indication of small cell lung cancer (SCLC) and for three patients complete treatment data was lacking. Of the remaining 115 patients, 62 received DLD cisplatin with concurrent chemo radiotherapy, 53 received a CHD cisplatin therapy. In total, 43 out of 115 patients did not complete treatment and follow-up due to various reasons (**Figure 2**). In the DLD treatment group, four patients did not continue treatment due to an adverse event, six were lost to follow-up, and one had another unknown reason for not reaching the end date. From the CHD treatment group there were 5 patients who did not continue cisplatin treatment due to progressive disease, 15 who did not continue treatment due to an adverse event, 2 refused further medication, 7 were lost to follow-up, and 3 had another unknown reason for not reaching the end date.

Patient characteristics of the total population, stratified by receiving DLD treatment or CHD treatment, are shown in **Table 1**. As compared to the CHD group, the DLD group was older, received less cumulative cisplatin dose, had an earlier cancer stage, had more comorbidities, and had higher baseline albumin levels. On the other side, the CHD treatment group had higher baseline eGFR, baseline phosphate levels, baseline numbers of leukocyte and platelets, and always received combination chemotherapy, including cisplatin combined with etoposide, gemcitabine, docetaxel, or pemetrexed.

**Table 1:** Patient’s characteristics for non-small lung cancer patients treated with cisplatin, split between the daily low-dose and the cyclic high-dose treatment group.

Characteristics		Total (n=115)		Daily low-dose (n=62)		Cyclic high-dose (n=53)		
Age at treatment, years (median; IQR)		65;	12	66;	11	65;		13
Gender	Male, N (%)	66	(57.39)	35;	(56.45)	31		(58.49)
Ethnicity, N (%)								
Caucasian		95	(82.61)	54	(87.1)	41		(77.36)
African		7	(6.09)	3	(4.84)	4		(7.55)
Arabic		8	(6.96)	5	(8.06)	3		(5.66)
Hispanic		4	(3.48)	0	(0)	4		(7.55)
Other		1	(0.87)	0	(0)	1		(1.89)
BMI, kg/m^2^ (median; IQR)		26.3;	6.48	26.66;	5.49	26;		6.05
BSA, m^2^ (median; IQR)		1.88;	0.26	1.94;	0.25	1.85;		0.75
Cancer Stage, N (%)								
IA		3	(2.61)	3	(4.84)	0		(0)
IB		1	(0.87)	1	(1.61)	0		(0)
IIA		1	(0.87)	0	(0)	1		(1.89)
IIB		11	(9.57)	5	(8.06)	6		(11.32)
IIIA		43	(37.39)	34	(54.84)	9		(16.98)
IIIB		21	(18.26)	17	(27.42)	4		(7.55)
IV		35	(30.43)	2	(3.23)	33		(62.26)
Histology of cancer, N (%)								
Adeno		63	(54.78)	29	(46.77)	34		(64.15)
Squamous		34	(29.57)	21	(33.87)	13		(24.53)
Large cell		15	(13.04)	10	(16.13)	5		(9.43)
Other		3	(2.61)	2	(3.23)	1		(1.89)
Karnofsky performance status (median; IQR)		90	(20)	90	(10)	85		(20)
WHO performance status, N (%)								
0		60	(52.17)	34	(54.84)	26		(49.06)
I		50	(43.48)	26	(41.94)	24		(45.28)
II		5	(4.35)	2	(3.22)	3		(5.66)
Chemotherapy, N (%)								
Cisplatin		62	(53.9)	62	(100)	–		–
Cisplatin/etoposide		2	(1.7)	–	–	2		(2.6)
Cisplatin/gemcitabine		11	(9.6)	–	–	11		(14.1)
Cisplatin/docetaxel		1	(0.9)	–	–	1		(1.3)
Cisplatin/pemetrexed		39	(33.9)	–	–	39		(50.0)
Cumulative cisplatin dose, mg/m^2^ (median; IQR)		144	(81)	144	(0)	225		(90)
Co-treatment with etoposide				NA		200		(80)
Co-treatment with gemcitabine						240		(100)
Co-treatment with docetaxel						150		(0)
Co-treatment with pemetrexed						225		(98.44)
Number of comorbidities, N (%)								
0		60	(52.2)	19	(30.6)	41		(77.4)
1		36	(31.3)	27	(43.5)	9		(17.0)
≥2		19	(16.5)	16	(25.8)	3		(5.7)
Baseline albumin, g/dl (median; IQR)		3.6;	1.4	4.1;	1.2	3.2		(1)
Baseline bilirubin, mg/dl (median; IQR)		0.29;	0.23	0.29;	0.18	0.29;		0.29
Baseline creatinine serum, mg/dl (mean (SD))		0.79	(0.19)	0.79	(0.18)	0.78		(0.17)
Baseline eGFR, ml/min/1.73 m^2^ (median; IQR)		92.85;	15.94	91.41;	19.38	94.71;		14.98
Baseline magnesium, mg/dl (median; IQR)		2;	0.04	2.00	0.18	2.00;		0
Baseline potassium, mEq/L (median; IQR)		4.4;	0.5	4.6	0.7	4.3		0.3
Baseline phosphate, mg/dl (mean (SD))		3.29	(0.51)	0.79	(0.18)	2.89		(0.30)
Baseline hemoglobin, g/dl (median; IQR)		13.5;	2.6	13.5	2.4	13.5		2.8
Baseline leukocyte, cells/mm^3^ (median; IQR)		9400;	4300	9211	4400	9500		4250
Baseline neutrophil, cells/mm^3^ (median; IQR)		6510;	3410	6525	3335	6510		3621
Baseline platelet, cells/mm^3^ (median; IQR)		324000;	164000	315000	163250	340000		162500

BMI, body mass index; BSA, body surface area; WHO, World Health Organization; IQR, interquartile range).

### Development of Myelotoxicity and Nephrotoxicity

When applying ≥grade 1 criteria as a cut-off point for developing toxic events, the most common toxic events reported during treatment were: anemia (60%), leukopenia (33.9%), neutropenia (31.3%), thrombocytopenia (27.8%), Cr-electrolyte nephrotoxicity (32.2%), SCr-AKI (12.2%), electrolyte abnormalities (23.5%), and chronic nephrotoxicity (58.3%). When we used ≥grade 2 as the cut-off point, toxic events were reduced to 23.5% for anemia, 20.0% for leukopenia, 13.9% for neutropenia, 7.0% for thrombocytopenia, 27.8% for Cr-electrolyte nephrotoxicity, 6.1% for SCr-AKI, and 20.9% for chronic nephrotoxicity while electrolyte normalities proportion did not change.

The proportions of ≥grade 1 toxicities were higher in DLD group except for anemia (53.2 *vs*. 67.9%), neutropenia (24.2 *vs*. 39.6%), and SCr-AKI (8.1 *vs*. 16.9%). However, the proportion of ≥grade 2 toxicities were lower in the DLD group except for thrombocytopenia (8.1 *vs*. 5.7%), Cr-electrolyte nephrotoxicity (38.7 *vs*. 15.1%), and electrolyte abnormalities (37.1 *vs*. 5.7%).

### Treatment Regimen as Predictor for Myelotoxicity and Nephrotoxicity

**Figure 3:**
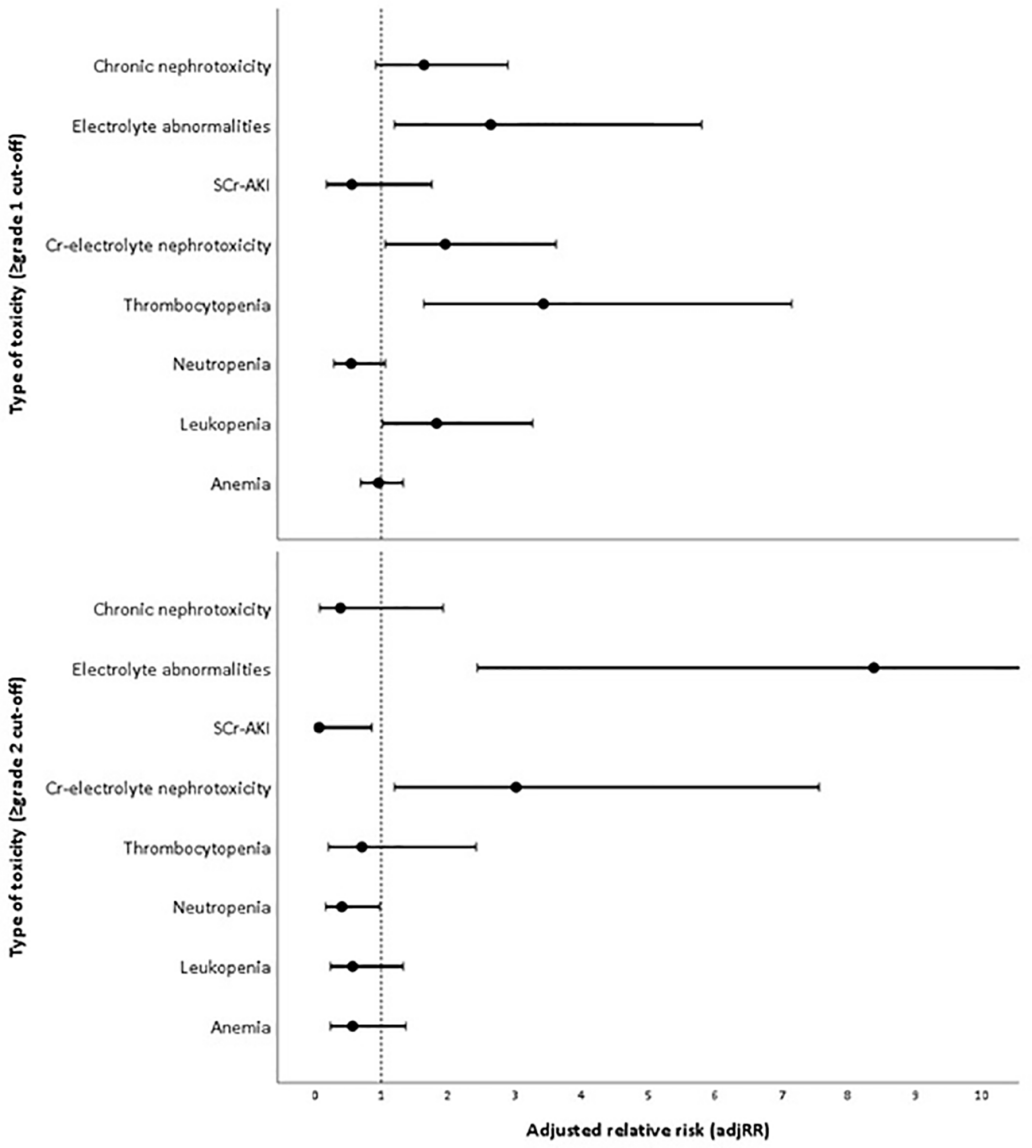
Adjusted risk of each toxicity in daily low-dose cisplatin (DLD) group compared to cyclic high-dose cisplatin (CHD) group in two cut-offs (≥grade 1 and ≥grade 2).

The proportion of patients that developed overall (≥grade 1) and moderate-to-severe (≥grade 2) adverse events for each treatment arm can be found in **Table 2**. The most frequent ≥grade 1 and ≥grade 2 adverse events in DLD group were chronic nephrotoxicity (64.5%) and Cr-electrolyte nephrotoxicity (38.7%). For CHD group, the most frequent ≥grade 1 and ≥grade 2 adverse events in DLD group were anemia (67.9 and 35.8%, respectively). The adjusted RRs for ≥grade 1 leukopenia [adjusted relative risk (adjRR) 1.83, 95% CI 1.02–3.27], and specifically thrombocytopenia (adjRR 3.43, 95% CI 1.64–7.15) were higher for the DLD group than the CHD group. As compared to the CHD group, the adjusted relative risk (adjRR) for ≥grade 2 neutropenia occurrence in the DLD group was lower (adjRR 0.41, 95% CI 0.17–0.98). Overall, as compared to the CHD group, the DLD group had a lower risk of having ≥grade 2 myelotoxicities (**Figure 3**). However, DLD had a higher risk with ≥grade 1 leukopenia and thrombocytopenia, suggesting that patients receiving the DLD regimen had a higher incidence of mild leukopenia and thrombocytopenia compared with patients receiving the CHD regimen.

**Table 2:** Occurrence of myelotoxicity and nephrotoxicity per treatment group and relative risk of daily low-dose cisplatin (DLD) compatred to cyclic high-dose cisplatin (CHD) for each toxicity.

Toxicities		DLD (n=62)		CHD (n=53)		adjRR^a,b^	95% CI	
Anemia	≥Grade 1	33	53.2%	36	67.9%	0.96	0.69	1.33
	≥Grade 2	8	12.9%	19	35.8%	0.57	0.24	1.37
Leukopenia	≥Grade 1	26	41.9%	13	24.5%	1.83	1.02	3.27
	≥Grade 2	11	17.7%	12	22.6%	0.57	0.24	1.33
Neutropenia	≥Grade 1	15	24.2%	21	39.6%	0.55	0.29	1.06
	≥Grade 2	5	8.1%	11	20.8%	0.41	0.17	0.98
Thrombocytopenia	≥Grade 1	25	40.3%	7	13.2%	3.43	1.64	7.15
	≥Grade 2	5	8.1%	3	5.7%	0.71	0.21	2.42
Cr-electrolyte nephrotoxicity	≥Grade 1	23	37.1%	14	26.4%	1.96	1.06	3.62
	≥Grade 2	24	38.7%	8	15.1%	3.02	1.20	7.56
SCr-AKI	≥Grade 1	5	8.1%	9	16.9%	0.56	0.18	1.76
	≥Grade 2	1	1.6%	6	11.3%	0.07	0.01	0.86
Electrolyte abnormalities	≥Grade 1	20	32.3%	7	13.2%	2.64	1.20	5.80
	≥Grade 2	23	37.1%	3	5.7%	8.38	2.44	28.81
Chronic nephrotoxicity	≥Grade 1	40	64.5%	27	50.9%	1.64	0.92	2.90
	≥Grade 2	9	14.5%	15	28.3%	0.39	0.08	1.93

Toxicity recorded as mild toxicity (grade≥1) and moderate-to-severe toxicity (grade ≥2).

a Modified Poisson regression was conducted by including adjustment for confounder and covariates. All outcomes were adjusted for age at treatment and gender except for chronic nephrotoxicity. Myelotoxicity outcome were adjusted for age at treatment, gender, number of comorbidities, and any of the following baseline disease states as defined by blood cell counts; anemia by hemoglobin, leukopenia by leukocytes, neutropenia by neutrophils, thrombocytopenia by platelets. Acute nephrotoxicity outcome was adjusted for number of comorbidities, baseline serum creatinine, albumin, magnesium, phosphate, and potassium while chronic nephrotoxicity was adjusted for baseline albumin and eGFR.

b CHD regimen was the reference group for each toxicity analysis.

The proportion of patients developing ≥grade 1 nephrotoxicity for the DLD and CHD group was 37.1–64.5% and 26.4–50.9% respectively, and the incidence of moderate-to-severe nephrotoxicity was 14.5–38.7% and 15.1–28.3% respectively. Adjusted risk for Cr-electrolyte nephrotoxicity, either using ≥grade 1 or ≥grade 2 cut-offs, were higher on DLD arm (adjRR 1.96, 95% CI 1.06–3.62 and adjRR 3.02, 95% CI 1.20–7.56). The risk of SCr-AKI was lower in DLD than in CHD arm (≥grade 1: adjRR 0.56, 95% CI 0.18–1.76; ≥grade 2: adjRR 0.07, 95% CI 0.01–0.86) when we differentiated between AKI-SCr and electrolyte abnormalities, suggesting that the risk on electrolyte abnormalities was substantially higher in the DLD than in the CHD arm. The adjusted risk for ≥grade 1 chronic nephrotoxicity was higher on DLD group as well (adjRR 1.64, 95% CI 0.92–2.90). However, the adjusted risk was lower when using ≥grade 2 cut-offs (adjRR 0.39, 95% CI 0.08–1.93). Overall, as compared to the CHD group, the DLD group had a higher risk on developing nephrotoxicities except for ≥grade 2 chronic nephrotoxicity (**Figure 3**).

**Figure 4:**
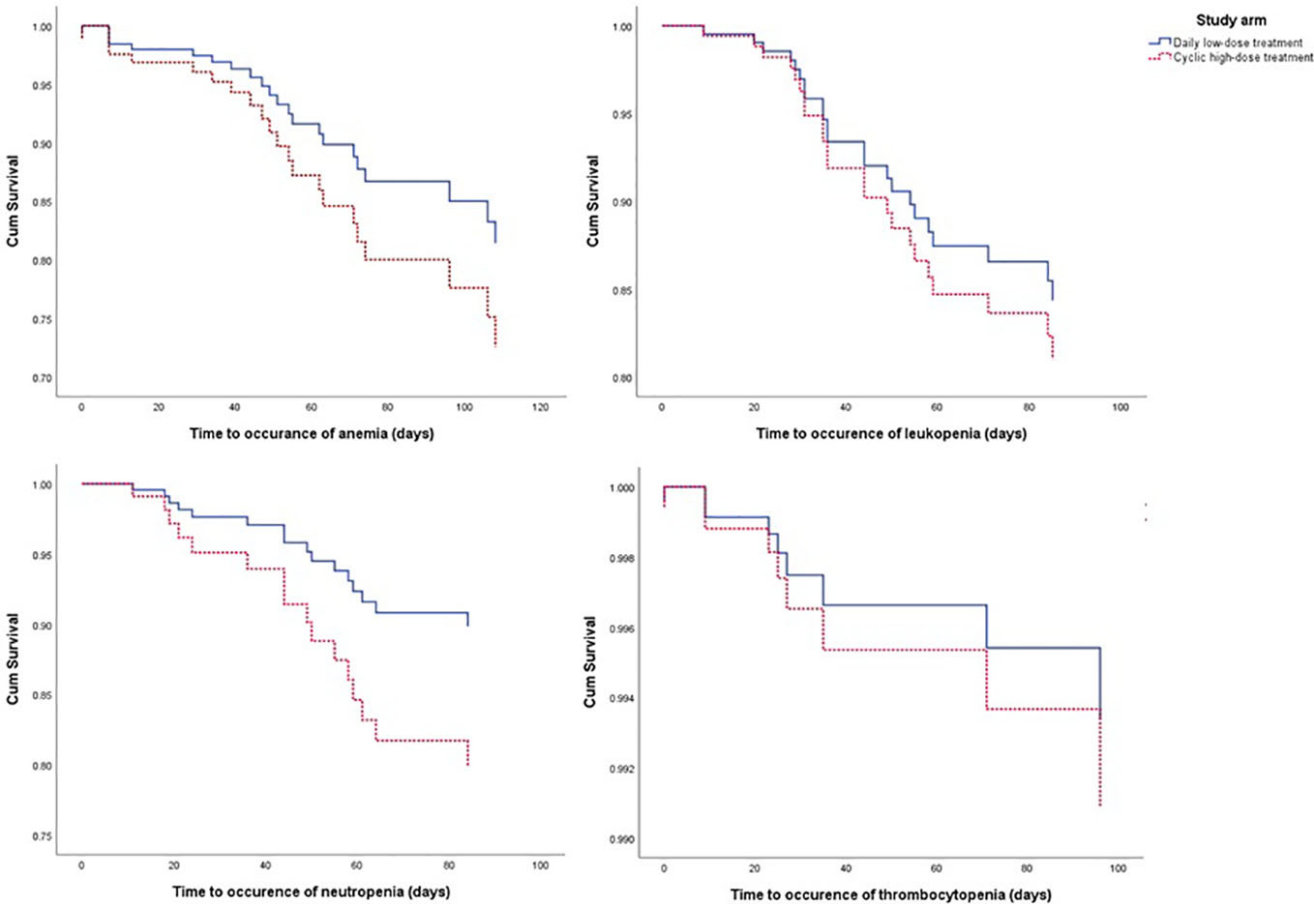
The Kaplan-Meier curve of daily low-dose cisplatin (DLD) and cyclic high-dose cisplatin (CHD) treatment group on ≥grade 2 myelotoxicity during 120 days of observation. Cum survival, cumulative survival.

**Figure 5:**
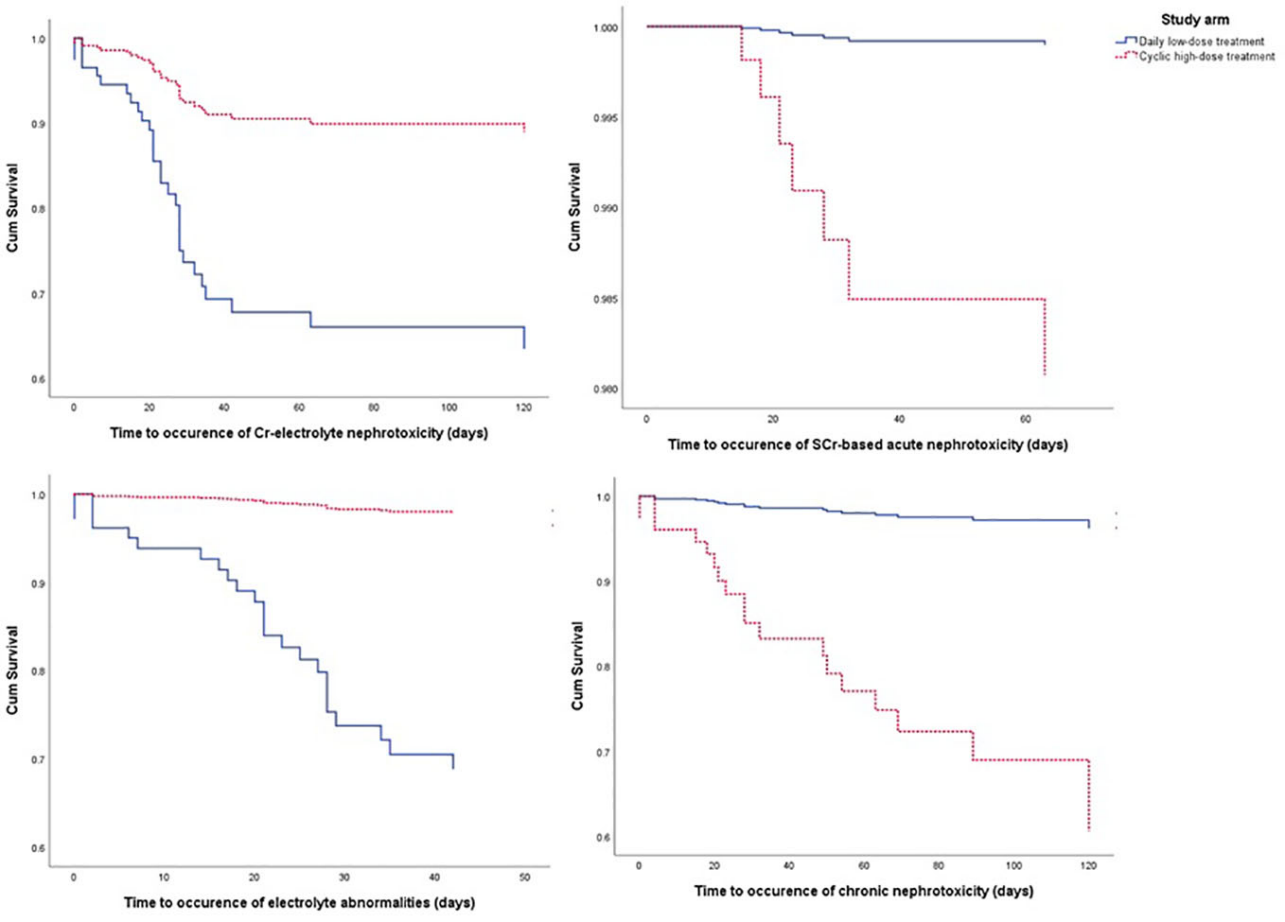
The Kaplan-Meier curve of daily low-dose cisplatin (DLD) and cyclic high-dose cisplatin (CHD) treatment group on ≥grade 2 nephrotoxicity and electrolyte abnormalities during 120 days of observation. Cum survival, cumulative survival.

From the Kaplan-Meier curve (**Figures 4** and **5**), we observed that the DLD group had a lower risk of ≥grade 2 toxicity than CHD except for Cr-electrolyte nephrotoxicity and electrolyte abnormalities. The results from time-to-event analysis also confirmed that patients from the DLD group had a lower hazard on having ≥grade 2 toxicity except for Cr-electrolyte nephrotoxicity [adjusted hazard ratio (adjHR) 3.90, 95% CI 1.35–11.23] and electrolyte abnormalities (adjHR 17.40, CI 95% 3.04–99.71) (**Table 3**). In addition, we found no statistically significant differences in occurrence of first toxicity before and after 120 days since the start of treatment in both treatment arms (**Table 4**) proofing that selecting 120 days cut-off for Cox proportional hazard model was appropriate.

**Table 3:** Hazard ratio of moderate-to-severe (≥grade 2) myelotoxicity and nephrotoxicity of daily low-dose cisplatin compared to cyclic high-dose cisplatin.

Adverse events	adjHR^a^	95% CI	
Anemia	0.64	0.20	2.03
Leukopenia	0.81	0.27	2.42
Neutropenia	0.48	0.14	1.65
Thrombocytopenia	0.72	0.04	12.20
Cr-electrolyte nephrotoxicity	3.90	1.35	11.23
SCr-AKI	0.05	0.01	0.56
Electrolyte abnormalities	17.40	3.04	99.71
Chronic nephrotoxicity	0.05	< 0.01	2.59

a Cox regression was conducted by including adjustment confounder and covariates mentioned at Methods section.

Cyclic high-dose was the reference category.

**Table 4:** Comparison of first toxicity events occurred before and after 120 days of treatment.

Adverse events occurred after 120 days of start of treatment	DLD		CHD	
Anemia, N (%)	0	0	0	0
Leukopenia, N (%)	0	0	0	0
Neutropenia, N (%)	0	0	0	0
Thrombocytopenia, N (%)	0	0	0	0
Cr-electrolyte nephrotoxicity, N (%)	3	10.7	2	12.5
SCr-AKI, N (%)	0	0	0	0
Electrolyte abnormalities, N (%)	0	0	0	0
Chronic nephrotoxicity, N (%)	1	11.1	1	6.7

As for the secondary endpoint, the reduction in eGFR from baseline was less in the DLD compared to the CHD group (β=8.49, 95% CI 1.86–15.13), indicating a potential protective effect with respect to the occurrence of chronic kidney disease (**Table 5**).

**Table 5:** Estimated glomerular filtration rate (eGFR) at baseline, at the end of chemotherapy, and 90 days after the last cisplatin administration in both treatment arms.

Time of eGFR assessment	Daily low-dose	Cyclic high-dose	B (95% CI)^a^
At baseline (n=115), ml/min/1.73 m^2^ (median; IQR)	89.56 ± 15.09	92.30 ± 14.94	8.49 (1.86-15.13)
At the end of chemotherapy (n=96), ml/min/1.73 m^2^ (median; IQR)	86.17 ± 18.86	75.97 ± 26.11	
90 days after the last cisplatin administration (n=65), ml/min/1.73 m^2^ (median; IQR)	84.73 ± 17.97	81.91 ± 21.45	

a Result from generalized estimating equations (GEE) test, correcting for number of comorbidity and baseline albumin.

## Discussion

Cisplatin may cause many types of toxicity, including myelotoxicity, acute, and chronic nephrotoxicity accompanied by electrolyte abnormalities (Hartmann and Lipp, 2003). In this study we compared the risk of myelotoxicity and nephrotoxicity between two cisplatin treatment regimens which differed in the height and frequency of dosing: the DLD and CHD regimen. We demonstrated that the DLD regimen led to higher risk on ≥grade 1 leukopenia, thrombocytopenia, Cr-electrolyte and chronic nephrotoxicity and electrolyte abnormalities but lower on risk on anemia, neutropenia, and SCr-AKI as compared to the CHD regimen. However, when we considered the more severe adverse drug reactions (≥grade 2), the DLD regimen led to lower risk on all toxicities except Cr-electrolyte nephrotoxicity and electrolyte abnormalities based on time-to-event analysis. Thus, both the high dosing frequency in the DLD regimen and the high dose in the CHD regimen affect the toxicity profile of cisplatin treatment.

Our findings are in line with a previous randomized controlled trial comparing sequential (CHD cisplatin plus gemcitabine) *versus* concurrent chemo-radiotherapy (DLD cisplatin plus radiotherapy) in 142 patients with inoperable NSCLC (Belderbos et al., 2007). Belderbos *et al*. demonstrated that the proportion of patients experiencing thrombocytopenia, leukopenia, and granulocytopenia (≥ grade 3) was smaller when treated with DLD compared to the CHD regimen. However, the occurrence of treatment-related kidney toxicity was not investigated. In contrast, a non-randomized trial in 131 Dutch patients showed slightly higher incidence of severe late toxicity in DLD group compared to CHD (27 *vs*. 23% respectively) (Uitterhoeve et al., 2007). However, the type of toxicity of both arms was not reported in detail in this study, making it difficult to compare different type of toxicity occurred in both regimen.

Compared to the CHD arm, we found that patients in the DLD arm were more likely to experience electrolyte imbalance but were less likely to develop nephrotoxicity (marked by high serum creatinine and low eGFR) compared to CHD arm. We could not find evidence from human or *in vitro* studies using human cell-lines explaining the underlying mechanisms. However, according to a preclinical study in rodents, this might be related to the observation of a low cleaved caspase-3 level as a measure of apoptosis, a low level of tubular necrosis, low interleukin (IL)-6 messenger RNA (mRNA) levels (a pro-inflammatory cytokine that has been found in kidney injury), but a significant increase in infiltrating macrophages who play role in repair response post-injury in a high-frequency low-dose cisplatin regimen (Sharp et al., 2016). To conclude, cell death through apoptosis and necrosis was lower in mice subjected to the high-frequency low-dose cisplatin regimen (Sharp et al., 2016). This is in line with the decreased risk of serum creatinine based nephrotoxicity and eGFR in the DLD group as demonstrated in this study.

We found that electrolyte abnormalities and acute kidney toxicity related with low serum electrolyte was more likely to appear in the DLD group. It is difficult to explain the correlation of renal function and electrolyte abnormalities in the context of this study as eGFR and AKI-SCr analysis showed opposite results. However, we hypothesize this could be due to the concomitant radiotherapy the DLD group received during cisplatin administration. Incidence of acute esophageal toxicity tend to be higher in the DLD arm compared to sequential chemoradiation or radiotherapy only because of sensitizing effect of cisplatin (Belderbos et al., 2005). A previous study at our institution reported that acute esophageal toxicity grade 1 occurred in 32% of the DLD patients, while ≥grade 2 esophageal toxicity occurred in 57% of the patients treated with DLD (Uyterlinde et al., 2013). Esophagitis is characterized by mucosal atrophy, ulceration, inflammation (Choy et al., 1999; Nijkamp et al., 2013), and edema, leading to pain when swallowing. Thus, esophagitis may cause weight loss and malnutrition in DLD treated patients (Uyterlinde et al., 2013) which will affect the electrolyte intake from food and fluids.

Overall, we observed a milder toxicity profile of DLD cisplatin treatment as compared to the CHD cisplatin treatment except for electrolyte abnormalities. This milder toxicity profile indicates a slow decline over time of the blood cell counts and eGFR. We hypothesize that due to the constant daily administration, the patients could not recover from the decline before the next dose and therefore a linear decline in blood cell counts and eGFR over time is observed. On the other hand, the more severe toxicity profile in the CHD group indicates a rapid decline of blood counts and eGFR in the CHD group. However, due to the cyclic regimen the patient will be able to recover before the next cisplatin dose is received. Thereby, during the CHD regimen the blood counts and eGFR will follow a cyclic pattern of decline and increase over time, relative to the cisplatin dose. Thus, the results indicate that the incidence and severity of toxicity is directly related to the pharmacokinetics of cisplatin (Farris et al., 1988).

Furthermore, we know from other studies that the response on DLD treatment in terms of survival is equal to CHD treatment, thus less cumulative cisplatin dose still leads to the same therapeutic efficacy (Milano et al., 1990; Takata et al., 2002; Belderbos et al., 2007). Moreover, concurrent radiotherapy in the DLD regimen has an comparable safety profile to other radiotherapies (Kappers et al., 2011; Chen et al., 2013). The DLD regimen is equally effective as the standard CHD regimen while having a more favorable toxicity profile except for electrolyte abnormalities and acute nephrotoxicity marked by low serum electrolyte. Thus, while our results have to be validated in a prospective study, they deliver novel insights in cisplatin regimens that could help with future optimization of treatment regimen prescriptions.

A strength of this study is that we were able to perform a retrospective head-to-head comparison of two treatment regimens commonly applied in the centers included, of which the population represented current clinical practice in the Netherlands.

Some limitations of our study need to be addressed. Selection bias might have occurred in this study: patients with multiple comorbidities and lower cancer stage are less likely to receive CHD cisplatin treatment and will receive DLD treatment or radiotherapy. Therefore, the incidence of toxicity caused by the CHD regimen could be underestimated. Risk of selection bias also occurs as we included patients from different hospitals. However, there were no significant differences in clinical characteristics between DLD-treated patients between both institutions except in performance status. Because of limited power, some clinically significant differences may not be identified as statistically significant in this study. In addition, the study was limited by missing data. Especially the data on electrolyte concentrations was very limited, as the measurement was not incorporated in the standard clinical procedure. This might have led to an underestimation of the incidence and grading of acute nephrotoxicity in this study. However, it is likely that the missing values were not random: for patients performing well, electrolytes would not be measured; for patients who are performing worse, suspicion may have led to measurement of electrolytes. Therefore, we assume that the underestimation of acute nephrotoxicity is minimal. In addition, we specified the outcome to myelotoxicity and nephrotoxicity since other cisplatin toxicities such as nausea, vomiting, ototoxicity, and neurotoxicity were not regularly assessed. Finally, different chemotherapy doublets were included within the CHD group. From subgroup analysis we found that patients receiving the CHD-gemcitabine combination have a higher risk of ≥grade 2 leukopenia, neutropenia, and chronic nephrotoxicity compared to CHD-pemetrexed combination, confirming the effect of different antineoplastic combination on the toxicity (**Table S2**). However, since the scope of the study was to compare DLD cisplatin treatment to CHD cisplatin treatment, the differences in doublet therapy do represent current clinical practice.

In conclusion, this study demonstrates a higher risk on overall (≥grade 1) toxicities in the DLD regimen, and a lower risk on moderate-to-severe (≥grade 2) toxicities in the DLD regimen, as compared to the CHD regimen. However, the risk of electrolyte abnormalities during DLD regimen should be closely monitored in daily basis during therapy so that adequate supportive care intervention can be given appropriately. Based on our findings, choice of the cisplatin dose should be based on the patients’ age, comorbidities as well as electrolyte status (Koning et al., 2010). Overall, the DLD regimen leads to equal therapeutic efficacy with a lower risk profile for moderate-to-severe toxicities, indicating that the DLD regimen could improve patients’ quality of life.

## Data Availability Statement

The datasets generated for this study are available on request to the corresponding author.

## Ethics Statement

The studies involving human participants were reviewed and approved by Medisch Ethische Toetsingscommissie, Academisch Medisch Centrum (AMC) Universiteit van Amsterdam. The patients/participants provided their written informed consent to participate in this study.

## Author Contributions 

Conceptualization: ZZ, JV, and A-HM-Z. Data curation: RK, JV, WU, ZZ, and PB. Formal analysis: ZZ, RK, and CL. Funding acquisition: ZZ, A-HM-Z. Investigation: ZZ, RK, JV, WU, CL, PB, RM, SV, and A-HM-Z. Methodology: ZZ, RK, JV, CL, PB, RM, SV, and A-HM-Z. Project administration: RK, RM, and A-HM-Z. Resources: PB, RM, SV and A-HM-Z. Supervision: PB, SV, RM, and A-HM-Z. Visualization: ZZ and RK. Writing—original draft: ZZ and RK. Writing—review and editing: ZZ, RK, JV, WU, CL, PB, RM, SV, and A-HM-Z. All authors contributed to the article and approved the submitted version.

## Funding

This work was supported by Indonesia Endowment Fund for Education (LPDP) Ministry of Finance, the Republic of Indonesia [as a part of ZZ’s Ph.D. project, grant no. 20161022049506, 2016]. The funder had no role in the design of the study; in the collection, analyses or interpretation of data; in the writing of the manuscript or in the decision to publish the results.

## Conflict of Interest

The authors declare that the research was conducted in the absence of any commercial or financial relationships that could be construed as a potential conflict of interest.

## Abbreviations

adjRR, adjusted relative risk; adjHR, adjusted hazard ratio; AKI, acute kidney injury; CHD, cyclic high-dose cisplatin; CKD, chronic kidney disease; CTCAE, common terminology criteria for adverse events; DLD, daily low-dose cisplatin; eGFR, estimated glomerular filtration rate; SCr, serum creatinine.
