# Supplementary material for: Comparison of Myelotoxicity and Nephrotoxicity Between Daily Low-Dose Cisplatin With Concurrent Radiation and Cyclic High-Dose Cisplatin in Non-Small Cell Lung Cancer Patients
Source: Front Pharmacol. 2020 Jun 26;11:975. doi: 10.3389/fphar.2020.00975 (PMC7332698; doi:10.3389/fphar.2020.00975)
Supplement: Supplementary file 1 [file Table_1.docx]

Comparison of Myelotoxicity and Nephrotoxicity Between Daily Low-Dose Cisplatin with Concurrent Radiation and Cyclic High-Dose Cisplatin in Non-Small Cell Lung Cancer Patients

Short title: Toxicity of daily low- vs cyclic high-dose cisplatin

Z Zazuli^1,2^, R Kos^1^, J D Veltman^1^, W Uyterlinde^3^, C Longo^1^ , P Baas^3^, R Masereeuw^4^, S J H Vijverberg^1^, A H Maitland-van der Zee^1*^

^1^Department of Respiratory Medicine, Amsterdam UMC, University of Amsterdam, Amsterdam, the Netherlands.

^2^Department of Pharmacology-Clinical Pharmacy, School of Pharmacy, Bandung Institute of Technology, Bandung, Indonesia.

^3^Department of Thoracic Oncology, The Netherlands Cancer Institute/Antoni van Leeuwenhoek Hospital, Amsterdam, The Netherlands.

^4^Division of Pharmacology, Utrecht Institute for Pharmaceutical Sciences, Utrecht, the Netherlands.

**Correspondence**: Anke-Hilse Maitland-van der Zee, Department of Respiratory Medicine, Amsterdam UMC location AMC, Room F5-227, Meibergdreef 9, 1105 AZ, Amsterdam, the Netherlands. E-mail: a.h.maitland@amsterdamumc.nl; Tel.: +31-(0)20-566-813

# Supplementary Material

| **Table S1.** Comparison of DLD-treated patients’ characteristics between Amsterdam UMC and NKI | | | | | | | | | |
| --- | --- | --- | --- | --- | --- | --- | --- | --- | --- |
| **Characteristics** | | | **All DLD patients**  **(n=62)** | | **From Amsterdam UMC**  **(n=25)** | | **From Netherlands Cancer Institute (n=37)** | | **p-value** |
| Age at treatment, years (median; IQR) | | | 66; | 11 | 68; | 12 | 65; | 14 | 0.064 |
| Gender | | Male, N (%) | 35; | (56.45) | 15 | (60) | 20 | (54.05) | 0.795 |
| Ethnicity, N (%) | | |  |  |  |  |  |  | 0.054 |
|  | Caucasian | | 54 | (87.1) | 19 | (76) | 35 | (94.59) |  |
|  | African | | 3 | (4.84) | 3 | (12) | 0 | (0) |  |
|  | Arabic | | 5 | (8.06) | 3 | (12) | 2 | (5.41) |  |
|  | Hispanic | | 0 | (0) | 0 | (0) | 0 | (0) |  |
|  | Other | | 0 | (0) | 0 | (0) | 0 | (0) |  |
| BMI, kg/m^2^ (median; IQR) | | | 26.66; | 5.49 | 25.71; | 8.39 | 28; | 5.06 | 0.282 |
| BSA, m^2^ (median; IQR) | | | 1.94; | 0.25 | 1.92; | 0.26 | 1.94; | 0.22 | 0.824 |
| Cancer Stage, N (%) | | |  |  |  |  |  |  | 0.061 |
|  | IA | | 3 | (4.84) | 3 | (12) | 0 | (0) |  |
|  | IB | | 1 | (1.61) | 0 | (0) | 1 | (2.7) |  |
|  | IIA | | 0 | (0) | 0 | (0) | 0 | (0) |  |
|  | IIB | | 5 | (8.06) | 3 | (12) | 2 | (5.41) |  |
|  | IIIA | | 34 | (54.84) | 10 | (40) | 24 | (64.86) |  |
|  | IIIB | | 17 | (27.42) | 7 | (28) | 10 | (27.03) |  |
|  | IV | | 2 | (3.23) | 2 | (8) | 0 | (0) |  |
| Histology of cancer, N (%) | | |  |  |  |  |  |  | 0.132 |
|  | Adeno | | 29 | (46.77) | 11 | (44) | 18 | (48.65) |  |
|  | Squamous | | 21 | (33.87) | 12 | (48) | 9 | (24.32) |  |
|  | Large cell | | 10 | (16.13) | 2 | (8) | 8 | (21.62) |  |
|  | Other | | 2 | (3.23) | 0 | (0) | 2 | (5.41) |  |
| Karnofsky Performance Status  (median; IQR) | | | 90 | (10) | 80; | 20 | 90; | 0 | <0.001* |
| WHO Performance Status, N (%) | | |  |  |  |  |  |  | 0.022* |
|  | 0 | | 34 | (54.84) | 9 | (36) | 25 | (67.57) |  |
|  | I | | 26 | (41.94) | 14 | (56) | 12 | (32.43) |  |
|  | II | | 2 | (3.22) | 2 | (8) | 0 | (0) |  |
| Cumulative Cisplatin Dose, mg/m^2^ (median; IQR) | | | 144 | (0) | 144 | (6) | 144; | 0 | 0.224 |
| Number of comorbidities, N (%) | | |  |  |  |  |  |  | 0.2 |
|  | 0 | | 19 | (30.6) | 7 | (28) | 12 | (32.43) |  |
|  | 1 | | 27 | (43.5) | 8 | (32) | 19 | (51.35) |  |
|  | ≥2 | | 16 | (25.8) | 10 | (40) | 6 | (16.22) |  |
| Abbreviations: BMI=Body Mass Index; BSA=Body Surface Area; WHO=World Health Organization, IQR: interquartile range) | | | | | | | | |  |

**Result of subgroup analysis between CHD-pemetrexed and CHD-gemcitabine**

- Difference in cisplatin cumulative dose between CHD-pemetrexed and CHD-gemcitabine didn’t alter the risk of myelotoxicity and nephrotoxicity in both groups. No significant differences in cisplatin cumulative dose between patient receiving CHD+pemetrexed and CHD-gemcitabine (median (IQR) = 225 (98.44); median (IQR) = 240 (100) p=0.165).
- Differences in antineoplastic combination (gemcitabine and pemetrexed) alter the risk of myelotoxicity and nephrotoxicity. CHD-gemcitabine combination has higher risk of ≥grade 2 leukopenia, neutropenia, and chronic nephrotoxicity than CHD-pemetrexed combination.

Table S2. Occurrence of myelotoxicity and nephrotoxicity per treatment group and relative risk of CHD+pemetrexed compared to CHD+gemcitabine for each toxicity. Toxicity recorded as mild toxicity (grade≥1) and moderate-to-severe toxicity (grade ≥2)

| **Toxicities** | | **CHD+pemetrexed**  **(n=39)** | | **CHD+gemcitabine**  **(n=11)** | | **adjRR^#^** | **95% CI** | |
| --- | --- | --- | --- | --- | --- | --- | --- | --- |
| Anemia | ≥Grade 1 | 25 | 64.1% | 8 | 72,7% | 1.10 | 0.69 | 1.75 |
|  | ≥Grade 2 | 11 | 28,2% | 6 | 54,5% | 1.91 | 0.88 | 4.14 |
| Leukopenia | ≥Grade 1 | 10 | 25.6% | 2 | 18,2% | 0.46 | 0.15 | 1.37 |
|  | ≥Grade 2 | 4 | 10,3% | 6 | 54,5% | 6.41 | 1.73 | 23.82 |
| Neutropenia | ≥Grade 1 | 14 | 35.9% | 5 | 45,5% | 1.19 | 0.52 | 2.73 |
|  | ≥Grade 2 | 5 | 12,8% | 5 | 45,5% | 3.41 | 1.31 | 8.92 |
| Thrombocytopenia | ≥Grade 1 | 5 | 12,8% | 2 | 18,2% | 0.70 | 0.10 | 4.76 |
|  | ≥Grade 2 | 0 | 0,0% | 3 | 27,3% | NA | NA | NA |
| Acute nephrotoxicity (combination of SCr- and electrolyte-based) | ≥Grade 1 | 10 | 25,6% | 2 | 18,2% | 0.76 | 0.25 | 2.33 |
|  | ≥Grade 2 | 7 | 17,9% | 1 | 9,1% | 0.65 | 0.08 | 5.23 |
| Acute nephrotoxicity (SCr-based) | ≥Grade 1 | 6 | 15.4% | 1 | 9.1% | 0.57 | 0.13 | 2.43 |
|  | ≥Grade 2 | 5 | 9.1% | 1 | 9.1% | 0.59 | 0.09 | 3.92 |
| Electrolyte abnormalities | ≥Grade 1 | 5 | 12.8% | 2 | 18.2% | 2.16 | 0.41 | 11.48 |
|  | ≥Grade 2 | 3 | 7.7% | 0 | 0% | NA | NA | NA |
| Chronic nephrotoxicity | ≥Grade 1 | 19 | 48,7% | 6 | 54,5% | 1.07 | 0.32 | 3.64 |
|  | ≥Grade 2 | 11 | 28,2% | 3 | 27,3% | 27.85 | 1.16 | 671 |

^#^ Modified Poisson regression was conducted by including adjustment for confounder and covariates. All outcomes were adjusted for age at treatment and gender except for chronic nephrotoxicity. Myelotoxicity outcome were adjusted for age at treatment, gender, number of comorbidities, and any of the following baseline disease states as defined by blood cell counts; anemia by hemoglobin, leukopenia by leukocytes, neutropenia by neutrophils, thrombocytopenia by platelets. Acute nephrotoxicity outcome was adjusted for number of comorbidities, baseline serum creatinine, albumin, magnesium, phosphate, and potassium while chronic nephrotoxicity was adjusted for baseline albumin and eGFR.

* CHD+pemetrexed regimen was the reference group for each toxicity analysis.
